# Spatial Patterns of Variation in Climatic Niche Breadths in Agamid Lizards

**DOI:** 10.3390/ani16071028

**Published:** 2026-03-27

**Authors:** Zhi-Wen Wang, Zheng-Yuan Fang, Xu Hu, Pan-Pan Zhu, Kai-Xu Si, Yu Du, Long-Hui Lin, Xia-Ming Zhu

**Affiliations:** 1 Herpetological Research Center, Hangzhou Normal University, Hangzhou 311121, China; wangzhiwen@stu.hznu.edu.cn (Z.-W.W.); 2023111010101@stu.hznu.edu.cn (Z.-Y.F.); 2025112010011@stu.hznu.edu.cn (P.-P.Z.); 2025112010038@stu.hznu.edu.cn (K.-X.S.); 2 Zhejiang Provincial Key Laboratory of Wetland Intelligent Monitoring and Ecological Restoration, Hangzhou Normal University, Hangzhou 311121, China; 3 College of Life and Environmental Sciences, Hangzhou Normal University, Hangzhou 311121, China; hu_xu2018@hznu.edu.cn; 4 Hainan Key Laboratory of Herpetological Research, College of Fisheries and Life Sciences, Hainan Tropical Ocean University, Sanya 572022, China; yudu@hntou.edu.cn

**Keywords:** Agamidae, temperature, precipitation, niche breadth, niche position

## Abstract

Climatic niche breadth is defined as the range of climatic conditions (e.g., temperature and precipitation) under which a species occurs. However, the relationship between niche breadth variation and climatic factors remains poorly studied, and existing results require more general testing. We studied spatial patterns of variation in climatic niche breadths in lizards of the family Agamidae and compared patterns within and across regions to see if they parallel or differ from each other using geo-referenced occurrence records, climatic data and phylogenetic comparative methods. We found that (1) species in warmer environments have narrower temperature niche breadths; (2) precipitation niche breadths are positively correlated with precipitation niche position, and also with temperature niche breadths; and (3) most of the variation in temperature niche breadths is explained by within-locality variation in climatic conditions, whereas most of the variation in precipitation niche breadths is explained by among-locality variation. The patterns of climatic niche breadth in agamids are consistent across regional and global scales, similar to those in other amphibians and reptiles. This suggests that this is a widespread phenomenon among ectothermic vertebrates.

## Simple Summary

Temperature and precipitation niche breadths are defined as the range of climatic conditions under which a species occurs, and they help determine where a species can persist across both space and time. The Agamidae, a diverse family of lizards, is widely distributed across a broad spectrum of climates, ranging from rainforests to deserts and warm to cold regions. Our results indicated that temperature niche breadth decreased with niche position, whereas precipitation niche breadth increased with niche position. Variation in temperature niche breadth stems mainly from local climatic conditions, while variation in precipitation niche breadth arises from differences among localities. Patterns of climatic niche breadth for agamids are consistent across regions and align with those previously reported for amphibians and reptiles.

## 1. Introduction

Climatic niche breadth is defined as the range of climatic conditions (e.g., temperature and precipitation) under which a species occurs. It helps determine where a species can persist across space and time and is therefore important for addressing a variety of research questions, including those related to species-level colonization, spread, and survival [1,2], as well as broad-scale patterns of community diversity, structure and dynamics [3,4,5]. For example, species characterized by a broad climatic niche breadth are capable of colonizing multiple habitat types [6] or are more tolerant of varied climatic conditions [7]. As a result, they are more likely to encounter suitable conditions across larger geographical areas compared to species with a narrow climatic niche breadth [8]. Species in the temperate regions may have a lower ability to respond to climate warming via temperature niche evolution than species living in tropics regions, since temperate species undergo high climatic seasonality, which tends to drive a faster rate of evolution in their temperature niche [9,10,11].

Macroecological research examines patterns of climatic niche across both global [12,13,14,15,16] and regional [17,18,19] scales. A central aim is to determine whether these patterns vary among species or taxa across different continents, or if they remain consistent, reflecting a unified global pattern. Despite its importance, the link between shifts in niche breadth and climatic factors has received insufficient attention, and the generalizability of current results remains to be tested. A synthesis of these studies yields the following key findings: first, tropical species typically exhibit narrower temperature niche breadths than their temperate or cold-region counterparts, while showing broader precipitation niche breadths [3,13,14,20]. Second, species characterized by broader climatic niches typically demonstrate greater fluctuations in their experienced climates (seasonal temperature and precipitation extremes) between localities [14,21,22]. Third, the primary driver of variation in climatic niche breadths is seasonal variation within localities, supplemented by a secondary yet significant contribution from variation across species’ distribution ranges [14,21,22].

**Figure 1:**
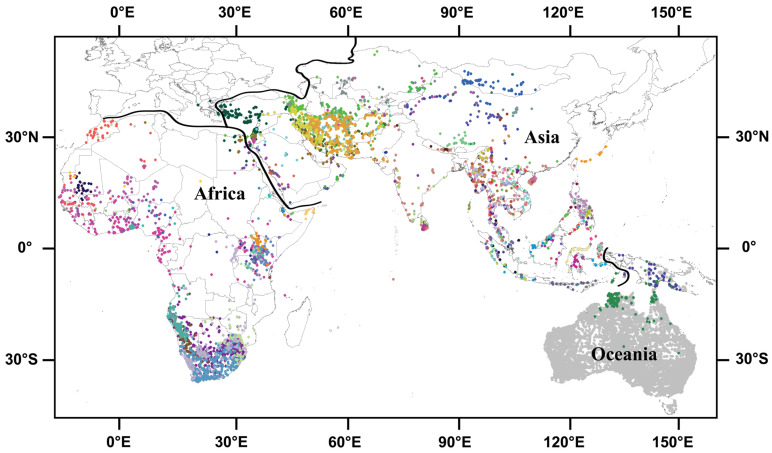
Map showing point localities of agamid lizards included in this study. Black solid lines indicate regional boundaries. Different colors represent different species, but the species endemic to Oceania are all in gray due to large sample size.

Here, we quantify temperature and precipitation niche breadths and evaluate how these patterns shift among closely related species across regions (continents). Focusing on the Agamidae, a diverse family of lizards with a wide geographical distribution spanning climates from rainforests to deserts and warm to cold regions (Figure 1) [23], we investigate how niche breadth patterns change on a global scale or regional scales. We initially compiled geo-referenced localities and temperature/precipitation data for all species within the phylogeny [24], and then address two main questions using phylogenetic comparative methods: (1) To what extent is a species’ climatic niche breadth along one dimension constrained by its position on that axis, and how might niche breadths be correlated across different dimensions? (2) Is interspecific variation in climatic niche breadth primarily attributable to local seasonal fluctuations (within-locality) or to spatial climatic heterogeneity across a species’ range (among-locality)?

## 2. Materials and Methods

Occurrence points for 212 species of agamids (Table S1) included in the phylogeny [24] were collected from published papers containing distribution information, and from public databases including Global Biodiversity Information Facility (http://www.gbif.org) and HerpNet/VertNet (http://www.vertnet.org). Duplicate occurrence points from different sources were deleted. Occurrence points were carefully checked against the known distribution range of each species (as documented in the Reptile Database [23]) to ensure all sampled localities were reasonable. Two methods were used in DIVA-GIS 7.5.0 [25] to detect outliers: (1) reverse jackknife, which was recommended for datasets with a normal distribution of values, such as those with many observations for each taxon [26]; and (2) 1.5 × interquartile range (1.5 IQR), which was recommended for datasets with a limited number of observations per taxon (e.g., *N* < 20 [25]). Of the 212 species, 204 occur only in one region (31 in Africa, 109 in Asia including the Middle East, Maluku Islands and Indonesian Archipelago, and 64 in Oceania including Australia, New Guinea and New Zealand), and eight in multiple regions [six in Africa and Asia, two in Asia and Oceania, and one (*Stellagama stellio*) in Africa, Asia and Europe] (Figure 1). For a species occurring in multiple regions, analyses of the species from each region were based only on its occurrence points for that region rather than its entire range. We extracted climatic variables for each occurrence point at ~1 km^2^ resolution from the WorldClim database (http://www.worldclim.org) using DIVA-GIS, and used data from 1950–2000 as a baseline [27]. We obtained climatic data from a mean of 242.9 localities per species, with a range of 2–3082 (Table S1).

We used the following six climatic variables to address the above questions: annual mean temperature (Bio1), maximum temperature of the warmest month (Bio5), minimum temperature of the coldest month (Bio6), annual precipitation (Bio12), precipitation of the wettest quarter (Bio16), and precipitation of the driest quarter (Bio17). We selected these climatic variables for the following reasons: (1) Bio1 and Bio12 are fundamental drivers of species distributions, reflecting energy and water availability; (2) temperature and precipitation niche positions were determined by the mean values of Bio1 and Bio12 across a species’ range, respectively; (3) temperature niche breadth (TNB) was calculated from the extreme values of Bio5 and Bio6; and (4) two indices of precipitation niche breadth (PNB) was calculated from those of Bio12, Bio16, and Bio17, the extreme values for Bio12, Bio16 and Bio17 [3,13,14,21,22].

For each species, TNB was calculated as the difference between the maximum Bio5 value and the minimum Bio6 value across all sampled localities. For PNB, we selected the following two indices. First, we calculated the range of Bio12 (maximum minus minimum) across a species’ range. Secondly, we subtracted the minimum value of Bio17 from the maximum value of Bio16 across a species’ range. The former index reflects spatial heterogeneity in precipitation, while the latter index reflects both spatial and seasonal precipitation variability.

We derived six metrics for each species with multiple regions: mean within-locality niche breadths (TNB_WL_ and PNB_WL_), mean ratios of within-locality to species-level niche breadth (TNBR_WL-S_ and PNBR_WL-S_), and niche position variances (TNPV and PNPV), for temperature and precipitation, respectively (Table S2). For each region, we calculated the TNB_WL_ and PNB_WL_ as the difference between Bio5 and Bio6 and between Bio16 and Bio17, respectively. For TNBR_WL-S_, we determined the ratio of TNB accounted by TNB_WL_ for each region, and then calculated the mean ratios for each species. For PNBR_WL-S_, we determined the ratio of PNB accounted by PNB_WL_ for each region, and then calculated the mean ratios for each species. For each region and globally, we calculated the mean values and ranges of TNBR_WL-S_ and PNBR_WL-S_. We evaluated the contribution of within-locality niche breadth by testing its relationship, via the calculated ratios, with species niche breadth. A positive relationship would indicate that within-locality variation substantially contributes to species niche breadth, while a negative relationship would suggest that among-locality variation is the primary driver. To assess the contribution of regional niche position variability to species niche breadth, we performed the following analysis. First, we calculated the regional niche position for each species as the midpoint between the relevant climatic variables: for temperature, this was the midpoint of Bio5 and Bio6; for precipitation, it was the midpoint of Bio16 and Bio17. We then quantified the variance of these midpoint values across all regions for each species. Finally, we examined whether a positive relationship exists between a species’ niche position variance and its niche breadth. Such a relationship would constitute evidence that among-locality variation in climatic conditions contributes to species niche breadth.

In order to account for the non-independence of data resulting from the shared evolutionary history of species, we analyzed data using phylogenetic generalized least squares regression (PGLS). To do that, we generated a phylogeny for 212 species using an agamid dataset (Appendix S1) extracted from a Squamata phylogeny comprising 4161 species [24]. The λ model was used for all PGLS analyses with branch lengths adjusted based on λ values estimated via maximum likelihood and values of kappa and delta fixed at 1. The λ model accounts for the estimated level of phylogenetic signal in the data, and this phylogenetic signal is what PGLS is designed to accommodate. In the first analysis, we examined the associations between niche breadth and niche position for both temperature and precipitation, as well as the correlation between TNB and PNB. In the second analysis, we assessed how species niche breadth relates to mean within-locality niche breadths (TNB_WL_, PNB_WL_), the mean ratios of within-locality to species niche breadth (TNBR_WL-S_, PNBR_WL-S_), and niche position variances (TNPV, PNPV). We performed all PGLS analyses in R 3.2.0 [28] with the package Caper 0.5 [29].

## 3. Results

**Figure 2:**
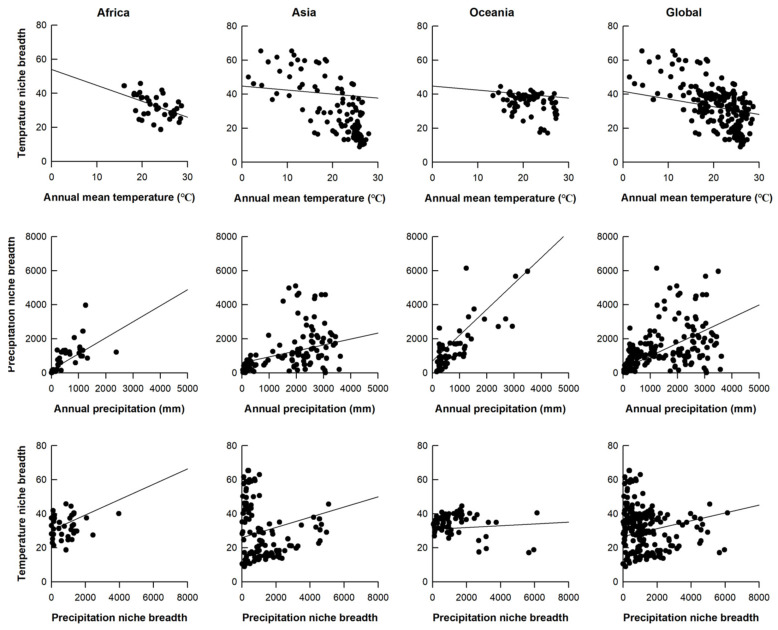
Relationships between TNB and annual mean temperature, between PNB and annual precipitation, and between TNB and PNB across regions and globally.

Globally, species experiencing higher mean Bio1 values in warmer regions generally possess a narrower TNB (*r*^2^ = 0.102, *p* < 0.0001; Table 1, Figure 2). Regionally, the negative relationship between TNB and Bio1 is significant in Africa, Asia and Oceania (Table 1, Figure 2). Globally, species inhabiting drier regions with low mean Bio12 tend to possess a narrower PNB (*r*^2^ = 0.154, *p* < 0.0001; Table 1, Figure 2). Regionally, the positive relationship between PNB and Bio12 is significant in Africa, Asia and Oceania (Table 1, Figure 2). Neither globally nor regionally is there a trade-off between TNB and PNB among species (Table 1). Instead, there is a positive relationship between TNB and PNB in Africa (*r*^2^ = 0.207, *p* < 0.01), Asia (*r*^2^ = 0.178, *p* < 0.0001) and globally (*r*^2^ = 0.123, *p* < 0.0001); the relationship in Oceania is non-significant (Table 1).

Globally, the mean TNBR_WL-S_ is 0.722, with species mean values ranging from 0.396 to 0.990 (Table 2). Regional mean values were highly consistent (0.719 to 0.733), with varied species mean values: Africa (0.396 to 0.983), Asia (0.415 to 0.990), and Oceania (0.467 to 0.939) (Table 2). Globally, the mean PNBR_WL-S_ is 0.478, with species mean values ranging from 0.042 to 0.980 (Table 2). Regional mean values for PNBR_WL-S_ are broadly similar, but noticeably lower in Oceania (0.350) and higher in Asia (0.563); species mean values range from 0.191 to 0.938 in Africa, 0.137 to 0.980 in Asia, and 0.042 to 0.793 in Oceania (Table 2).

The positive relationship between TNB_WL_ and TNB is significant for all regions and globally, and so is the positive relationship between PNB_WL_ and PNB (Table 3). The negative relationship between TNBR_WL-S_ and TNB is significant for all regions and globally (Table 3). The negative relationship between PNBR_WL-S_ and PNB is significant for Asia (*r*^2^ = 0.193, *p* < 0.0001) and globally (*r*^2^ = 0.120, *p* < 0.0001) but not for Africa (*r*^2^ = 0.028, *p* = 0.322) and Oceania (*r*^2^ = 0.040, *p* = 0.108) where the relationship is marginally non-significant (Table 3). The positive relationship between TNPV and TNB is significant for all regions and globally, and so is the positive relationship between PNPV and PNB (Table 3).

## 4. Discussion

We observed similar macroecological patterns across different regions that parallel global patterns. These patterns are generally consistent with those reported for amphibians and other lizards [13,14,20,21,30], with only minor variation in relationships (patterns) among regions. Temperature and precipitation niche breadths show a positive correlation globally in agamids, whereas in Oceania, this pattern breaks down and becomes statistically non-significant. Precipitation niche breadth is significantly and positively related to niche position globally in agamid lizards, lacertid lizards [30] and amphibians [13] but not in varanid lizards [14].

The consistency observed in macroecological patterns likely stems from a shared response to climatic variation across regions. A wide variety of biomes are represented across all four regions, including ecosystems ranging from deserts to rainforests and spanning warm to cold climatic regimes (Figure 1) and, though not found in regions with severely cold climates, agamid lizards occur in most of these environments. Therefore, it is perhaps expected that agamid lizards in different regions exhibit distinct patterns of climatic niche breadth, given the underlying variation in regional climatic conditions. Our results parallel those of previous studies on amphibians and other lizard species [13,14,21], supporting two conclusions: (1) patterns across regions generally parallel each other and reflect the global trend, and (2) similar trends are shared across different taxonomic groups of ectothermic vertebrates.

Our analysis corroborates the relationship between climatic niche breadth and position reported in prior studies. Specifically, a significant negative relationship between TNB and Bio1 was found at both global and regional scales. The pattern can be explained by an increase in temperature seasonality at higher latitudes, where mean annual temperatures are lower. Even at a single locality, this heightened seasonality enables species to occupy broader thermal niche breadths [21,31,32]. Consistent with this pattern, studies on hylid frogs, plethodontid salamanders, phrynosomatid lizards [21], and lacertid lizards [30] have all reported a positive relationship between thermal niche breadth and latitude. Similarly, our analysis revealed a positive association between position and niche breadth in precipitation (indexed by annual precipitation), consistent with reports from North American phrynosomatid lizards [20] and global-scale amphibian studies [13]. High precipitation seasonality at a lower latitude may lead to a wider PNB. Our finding supports the hypothesis that species from drier climates are more specialized, whereas those from mesic environments show broader precipitation niches—likely as a result of weaker selective constraints [13]. In a word, as predicted by Janzen’s hypothesis [32] and Rapoport’s rule [33], species inhabiting regions with high climatic seasonality tend to exhibit broader thermal tolerance and, consequently, a wider climatic niche breadth.

The hypothesized trade-off between precipitation and temperature niche axes posits that temperate species possess a wide temperature niche and a narrow precipitation niche, while tropical species display the reverse—a narrow temperature niche and a wide precipitation niche [13,14,20,32]. Instead, we found a positive relationship between niche widths on these two axes, as found regionally in phrynosomatid lizards [21] and globally in amphibians [13] and varanid lizards [14]. This may be because species restricted to narrow geographic distributions (e.g., island or desert species) due to their narrow tolerance along one niche axis (e.g., temperature) might consequently experience similarly limited conditions along another axis (e.g., precipitation). For example, *Agama finchi*, *A. kaimosae*, and *A. mwanzae*—which inhabit inland cliffs and mountain peaks of East Africa [34]—exhibit narrow niche breadths for both temperature and precipitation. In contrast, *A. agama*, distributed across West and Central Africa, possesses broad temperature and precipitation niche breadths, reflecting the stable and humid conditions of tropical rainforest climates. For agamids in Oceania, the relationship between temperature and precipitation niche axes was not significant. For example, genus *Hypsilurus* (*H*. *bruijnii*, *H*. *modestus*, *H*. *papuensis*) and *Lophosaurus* (*L*. *boydii*, *L*. *dilophus*) lizards across the Maluku Islands and Papua New Guinea display broad precipitation niche breadths, a pattern that aligns with the humid and climatically stable conditions of tropical rainforests. Most lizards distributed in Australia inhabit tropical grassland and tropical desert climates, both characterized by narrow precipitation niche widths, while their temperature niche widths vary.

Our results corroborate patterns observed in salamanders, frogs, lizards, and snakes [13,14,22], indicating that species-level temperature niche breadths are thus largely explained by within-locality variation, with only a minor contribution from spatial variation in thermal conditions across a species’ range. Across clades, the values remain strikingly similar. For agamids, within-locality variation in TNBs explained approximately 72% of the variation, and values for plethodonitids, hylids, phrynosomatids, varanids and elapids are 80%, 73%, 76%, 73% and 75%, respectively [13,14,22]. In agamids and elapids, among-locality variation is of greater importance for PNBs: within-locality variation explains only 48% and 43% [22] of species-level PNBs, respectively. These proportions are noticeably lower than those reported for plethodontids (63%), hylids (57%), phrynosomatids (59%), and varanids (57%) [13,14]. Consequently, within-locality niche breadth determines the species-level climatic niche breadth (at least for temperature), rendering variation between localities relatively unimportant. This implies that temporal variation within each locality, rather than spatial climatic variation across the species’ range, accounts for its climatic niche breadth.

## 5. Conclusions

Our findings reveal that climatic niche breadth patterns in agamids are consistent across regional and global scales, aligning with those reported for other ectothermic groups including amphibians (~38% of described species globally), phrynosomatid and varanid lizards, and elapid snakes. Species inhabiting warmer climates, characterized by a higher mean temperature niche position, tend to exhibit a narrower temperature niche breadth. In contrast, species in more humid climates, with a higher mean precipitation niche position, generally display a broader precipitation niche breadth. A positive relationship between species-level climatic niche breadth and within-locality niche breadth and within-locality variation explains most variation in temperature niche breadths. Our results provide evidence that these patterns may be widespread among ectothermic vertebrates.

## Figures and Tables

**Table 1:** PGLS results of the relationship between TNB and position (Bio1), between PNB and position (Bio12), and between TNB and PNB. *N* is the number of species.

		*N*	*λ*	*r* ^2^	*p* Value	Slope	Intercept
TNB vs. Bio1	Africa	37	0	0.247	<0.01	−0.935	54.06
	Asia	117	0.810	0.083	<0.01	−0.661	44.79
	Oceania	66	0.598	0.098	0.010	−0.452	41.68
	Global	212	0.827	0.102	<0.0001	−0.628	44.41
PNB vs. Bio12	Africa	37	0.091	0.363	<0.0001	0.940	190.50
	Asia	117	0.333	0.043	0.024	0.350	591.73
	Oceania	66	0.924	0.417	<0.0001	1.518	702.78
	Global	212	0.597	0.154	<0.0001	0.750	241.28
TNB vs. PNB	Africa	37	0.880	0.207	<0.01	0.0045	30.37
	Asia	117	0.914	0.178	<0.0001	0.0030	25.94
	Oceania	66	0.714	0.011	0.392	0.0005	30.84
	Global	212	0.934	0.123	<0.0001	0.0023	26.67

**Table 2:** Mean and range of the ratios of within-locality niche breadth to species niche breadth for temperature (TNBR_WL-S_) and precipitation (PNBR_WL-S_), summarized by region and globally.

	*N*	TNBR_WL-S_	PNBR_WL-S_
Africa	37	0.719 (0.396–0.983)	0.462 (0.191–0.938)
Asia	117	0.727 (0.415–0.990)	0.563 (0.137–0.980)
Oceania	66	0.733 (0.467–0.939)	0.350 (0.042–0.793)
Global	212	0.722 (0.396–0.990)	0.478 (0.042–0.980)

**Table 3:** PGLS results for the relationships between species niche breadth and three predictors: (i) mean within-locality breadths (TNB_WL_, PNB_WL_); (ii) mean ratios of within-locality to species-level breadth (TNBR_WL-S_, PNBR_WL-S_); and (iii) niche position variances (TNPV, PNPV), for both temperature and precipitation.

		*λ*	*r* ^2^	*p* Value	Slope	Intercept
TNB_WL_ vs. TNB	Africa	0.764	0.499	<0.0001	0.425	11.60
	Asia	0.750	0.620	<0.0001	0.486	7.52
	Oceania	0.839	0.235	<0.0001	0.354	10.23
	Global	0.842	0.492	<0.0001	0.449	8.74
PNB_WL_ vs. PNB	Africa	0	0.694	<0.0001	0.395	19.96
	Asia	0.661	0.493	<0.0001	0.265	259.67
	Oceania	1.000	0.309	<0.0001	0.239	103.88
	Global	0.763	0.436	<0.0001	0.265	223.79
TNBR_WL-S_ vs. TNB	Africa	0.893	0.461	<0.0001	−0.0108	1.14
	Asia	0.611	0.203	<0.0001	−0.0064	0.94
	Oceania	0.897	0.258	<0.0001	−0.0106	1.01
	Global	0.803	0.212	<0.0001	−0.0075	0.97
PNBR_WL-S_ vs. PNB	Africa	0	0.028	0.322	0.0000	0.49
	Asia	0.513	0.193	<0.0001	−0.0001	0.68
	Oceania	0.568	0.040	0.108	−0.0001	0.43
	Global	0.631	0.120	<0.0001	−0.0001	0.62
TNPV vs. TNB	Africa	0.115	0.142	0.022	0.258	−3.98
	Asia	0	0.354	<0.0001	0.221	−1.22
	Oceania	0.646	0.132	<0.01	0.186	−2.86
	Global	0.269	0.210	<0.0001	0.253	−3.28
PNPV vs. PNB	Africa	0	0.752	<0.0001	16.313	−2443.59
	Asia	0.646	0.764	<0.0001	39.080	−17,111.14
	Oceania	1.000	0.180	<0.001	12.884	6526.70
	Global	0.657	0.652	<0.0001	34.030	−11,696.47

## Data Availability

The original contributions presented in the study are included in the article/Supplementary Materials; further inquiries can be directed to the corresponding authors.

## Supplementary Materials

The following supporting information can be downloaded at: https://www.mdpi.com/article/10.3390/ani16071028/s1, Table S1: The number of occurrence points per species and its distribution between regions; Table S2: Summary of data on the number of geo-referenced localities per species (*N*), species niche breadths (TNB, PNB), species mean values for Bio1 and Bio12, within-locality niche breadths (TNB_WL_, PNB_WL_), the ratios of within-locality niche breadth to species niche breadth (TNBR_WL-S_, PNBR_WL-S_), and niche position variances (TNPV, PNPV) for both temperature and precipitation. Appendix S1: A phylogeny for 212 species using an agamid dataset extracted from Pyron et al. 2013 [24].

## Abbreviations

The following abbreviations are used in this manuscript:

TNB	Temperature niche breadth
PNB	Precipitation niche breadth
TNB_WL_	Within-locality temperature niche breadth
PNB_WL_	Within-locality precipitation niche breadth
TNBR_WL-S_	The ratios of within-locality niche breadth to species niche breadth for temperature
PNBR_WL-S_	The ratios of within-locality niche breadth to species niche breadth for precipitation
TNPV	The niche position variances for temperature
PNPV	The niche position variances for precipitation
