# Supplementary material for: Spatial Patterns of Variation in Climatic Niche Breadths in Agamid Lizards
Source: Animals (Basel). 2026 Mar 27;16(7):1028. doi: 10.3390/ani16071028 (PMC13072087; doi:10.3390/ani16071028)
Supplement: Supplementary file 1 [file animals-16-01028-s001.zip › Table S1.pdf]

Table S1. The number of occurrence points per species and its distribution between regions.

Species	Region(s)	Africa	Asia	Europe	Oceania
Acanthocercus atricollis	1	415			
Acanthosaura armata	1		6		
Acanthosaura capra	1		5		
Acanthosaura crucigera	1		24		
Acanthosaura lepidogaster	1		80		
Agama aculeata	1	523			
Agama agama	1	321			
Agama anchietae	1	251			
Agama armata	1	133			
Agama atra	1	600			
Agama boueti	1	65			
Agama boulengeri	1	31			
Agama caudospinosa	1	16			
Agama doriae	1	16			
Agama finchi	1	7			
Agama gracilimembris	1	10			
Agama hispida	1	140			
Agama impalearis	1	56			
Agama insularis	1	3			
Agama kaimosae	1	7			
Agama lionotus	1	64			
Agama mwanzae	1	22			
Agama paragama	1	11			
Agama planiceps	1	200			
Agama rueppelli	1	33			
Agama sankaranica	1	44			
Agama spinosa	1	10			
Agama weidholzi	1	28			
Amphibolurus muricatus	1				2512
Amphibolurus norrisi	1				259
Aphaniotis fusca	1		12		
Bronchocela cristatella	1		168		
Bufoniceps laungwalaensis	1		2		
Calotes calotes	1		21		
Calotes ceylonensis	1		6		
Calotes chincollium	1		80		
Calotes emma	1		152		
Calotes htunwini	1		45		
Calotes irawadi	1		70		
Calotes liocephalus	1		4		
Calotes liolepis	1		20		
Calotes mystaceus	1		288		

Calotes nigrilabris	1	4	
Calotes versicolor	1	653	
Ceratophora aspera	1	8	
Ceratophora erdeleni	1	5	
Ceratophora karu	1	5	
Ceratophora stoddartii	1	9	
Chelosania brunnea	1		66
Chlamydosaurus kingii	1		650
Cophotis ceylanica	1	7	
Cophotis dumbara	1	7	
Coryphophylax subcristatus	1	6	
Ctenophorus adelaidensis	1		143
Ctenophorus caudicinctus	1		1529
Ctenophorus clayi	1		156
Ctenophorus cristatus	1		737
Ctenophorus decresii	1		370
Ctenophorus femoralis	1		116
Ctenophorus fionni	1		314
Ctenophorus fordi	1		1339
Ctenophorus gibba	1		135
Ctenophorus isolepis	1		2089
Ctenophorus maculatus	1		484
Ctenophorus maculosus	1		64
Ctenophorus mckenziei	1		28
Ctenophorus nuchalis	1		2813
Ctenophorus ornatus	1		263
Ctenophorus pictus	1		1882
Ctenophorus reticulatus	1		780
Ctenophorus rufescens	1		73
Ctenophorus salinarum	1		173
Ctenophorus scutulatus	1		351
Ctenophorus tjantjalka	1		50
Ctenophorus vadnappa	1		194
Diporiphora albilabris	1		226
Diporiphora amphiboluroides	1		70
Diporiphora arnhemica	1		95
Diporiphora australis	1		407
Diporiphora bennettii	1		346
Diporiphora bilineata	1		1966
Diporiphora lalliae	1		334
Diporiphora linga	1		75
Diporiphora magna	1		937
Diporiphora nobbi	1		1500
Diporiphora pindan	1		139

Diporiphora reginae	1		21
Diporiphora superba	1		35
Diporiphora valens	1		38
Diporiphora winneckei	1		375
Draco beccarii	1	120	
Draco biaro	1	6	
Draco bimaculatus	1	16	
Draco blanfordii	1	38	
Draco bourouniensis	1	2	
Draco caerulhians	1	10	
Draco cornutus	1	8	
Draco cyanopterus	1	5	
Draco dussumieri	1	2	
Draco fimbriatus	1	21	
Draco guentheri	1	6	
Draco haematopogon	1	32	
Draco indochinensis	1	6	
Draco lineatus	1	12	
Draco maculatus	1	68	
Draco maximus	1	9	
Draco melanopogon	1	79	
Draco mindanensis	1	6	
Draco obscurus	1	54	
Draco ornatus	1	8	
Draco palawanensis	1	11	
Draco quadrasi	1	5	
Draco quinquefasciatus	1	25	
Draco reticulatus	1	6	
Draco rhytisma	1	8	
Draco spilonotus	1	46	
Draco spilopterus	1	100	
Draco taeniopterus	1	35	
Draco timorensis	1	4	
Draco volans	1	43	
Gonocephalus chamaeleontinus	1	19	
Gonocephalus grandis	1	39	
Gonocephalus kuhlii	1	4	
Gowidon longirostris	1		1192
Gowidon temporalis	2	5	527
Hydrosaurus amboinensis	1	12	
Hypsilurus bruijnii	1		8
Hypsilurus modestus	1		121
Hypsilurus nigrigularis	1		31
Hypsilurus papuensis	1		59

Intellagama lesueurii	1		3082
Japalura flaviceps	1	4	
Japalura polygonata	1	35	
Japalura splendida	1	8	
Japalura tricarinata	1	2	
Laudakia nupta	1	251	
Laudakia sacra	1	3	
Laudakia tuberculata	1	6	
Leiolepis belliana	1	64	
Leiolepis guentherpetersi	1	3	
Leiolepis guttata	1	2	
Leiolepis reevesii	1	5	
Lophognathus gilberti	1		1871
Lophosaurus boydii	1		59
Lophosaurus dilophus	2	2	62
Lophosaurus spinipes	1		317
Lyriocephalus scutatus	1	3	
Mantheyus phuwuanensis	1	8	
Moloch horridus	1		939
Otocryptis wiegmanni	1	8	
Paralaudakia caucasia	1	290	
Paralaudakia erythrogaster	1	18	
Paralaudakia himalayana	1	7	
Paralaudakia lehmanni	1	4	
Paralaudakia microlepis	1	41	
Paralaudakia stoliczkana	1	15	
Phoxophrys nigrilabris	1	9	
Phrynocephalus axillaris	1	27	
Phrynocephalus forsythii	1	10	
Phrynocephalus guttatus	1	27	
Phrynocephalus helioscopus	1	81	
Phrynocephalus interscapularis	1	22	
Phrynocephalus mystaceus	1	46	
Phrynocephalus przewalskii	1	13	
Phrynocephalus putjatai	1	6	
Phrynocephalus raddei	1	9	
Phrynocephalus scutellatus	1	197	
Phrynocephalus theobaldi	1	22	
Phrynocephalus versicolor	1	85	
Phrynocephalus vlangalii	1	25	
Physignathus cocincinus	1	32	
Pogona barbata	1		2571
Pogona henrylawsoni	1		44
Pogona minima	1		21

Pogona minor	1				1421
Pogona nullarbor	1				72
Pogona vitticeps	1				2439
Pseudocalotes brevipes	1		5		
Pseudocalotes flavigula	1		5		
Pseudocalotes kakhienensis	1		8		
Pseudotrapelus sinaitus	2	7	127		
Ptyctolaemus collicristatus	1		18		
Ptyctolaemus gularis	1		22		
Rankinia diemensis	1				997
Saara asmussi	1		22		
Saara hardwickii	1		5		
Saara loricata	1		42		
Sitana ponticeriana	1		14		
Stellagama stellio	3	11	461	9	
Trapelus agilis	1		432		
Trapelus flavimaculatus	1		14		
Trapelus mutabilis	2	3	44		
Trapelus ruderatus	1		148		
Trapelus sanguinolentus	1		47		
Trapelus savignii	2	2	31		
Tympanocryptis cephalus	1				225
Tympanocryptis intima	1				458
Tympanocryptis lineata	1				840
Tympanocryptis pinguicolla	1				72
Tympanocryptis tetraporophora	1				1117
Tympanocryptis uniformis	1				7
Uromastyx acanthinura	1	6			
Uromastyx aegyptia	2	10	224		
Uromastyx benti	1		3		
Uromastyx dispar	1	6			
Uromastyx geyri	1	9			
Uromastyx macfadyeni	1	3			
Uromastyx ocellata	1	5			
Uromastyx ornata	2	2	92		
Uromastyx princeps	1	16			
Uromastyx thomasi	1		9		
Xenagama taylori	1	3			
