# Supplementary material for: Spatial Patterns of Variation in Climatic Niche Breadths in Agamid Lizards
Source: Animals (Basel). 2026 Mar 27;16(7):1028. doi: 10.3390/ani16071028 (PMC13072087; doi:10.3390/ani16071028)
Supplement: Supplementary file 1 [file animals-16-01028-s001.zip › Table S2.pdf]

Table S2. Summary of data on the number of geo-referenced localities per species (*N*), species niche breadths (TNB, PNB), species mean values for Bio1, Bio12, within-locality niche breadths (TNB_{WL}, PNB_{WL}), the ratios of within-locality niche breadth to species niche breadth ($TNBR_{WL\text{-}S}$, $PNBR_{WL\text{-}S}$), and niche position variances (TNPV, PNPV) for both temperature and precipitation.

Speices	*N*	TNB	PNB[1]	PNB[2]	Bio1	Bio12	TNB_{WL}	PNB_{WL}	$TNBR_{WL\text{-}S}$	$PNBR_{WL\text{-}S}$	TNPV	PNPV
Acanthocercus atricollis	415	37.5	2065	1134	20.0	845.5	21.4	327.1	0.571	0.288	7.31	6052.3
Acanthosaura armata	6	17	1444	1475	25.6	2629.3	12.6	877.7	0.743	0.595	2.27	19422.8
Acanthosaura capra	5	22.2	1164	1379	21.8	2091.2	17.0	996.6	0.765	0.723	5.42	16178.1
Acanthosaura crucigera	24	22.6	4506	3697	25.6	2683.4	15.6	1403.3	0.692	0.380	2.76	129899.4
Acanthosaura lepidogaster	80	37	4558	3710	22.3	2029.8	19.5	1141.0	0.527	0.308	9.43	67009.5
Agama aculeata	523	39.9	1323	640	18.2	510.2	26.9	236.9	0.675	0.370	5.58	4959.8
Agama agama	321	40.1	3967	2826	24.6	1255.3	15.9	538.6	0.396	0.191	9.02	43986.4
Agama anchietae	251	40.4	1327	650	19.5	211.5	23.9	131.4	0.592	0.202	3.43	2238.3
Agama armata	133	37.4	1096	770	19.8	680.5	22.9	317.9	0.613	0.413	8.51	3724.7
Agama atra	600	44.4	1155	603	16.0	517.5	24.5	175.7	0.551	0.291	4.99	6380.0
Agama boueti	65	34.9	473	421	28.0	242.9	26.1	203.8	0.749	0.484	2.21	3300.7
Agama boulengeri	31	32.6	764	608	28.7	228.8	27.2	185.7	0.834	0.305	0.68	6326.5
Agama caudospinosa	16	29.8	1503	881	18.5	1042.2	17.6	318.6	0.591	0.362	10.86	14789.5
Agama doriae	16	30.6	1151	1087	23.2	1020.6	19.6	557.1	0.639	0.513	10.23	12622.9
Agama finchi	7	18.7	859	568	24.1	1323.4	15.0	339.9	0.803	0.598	1.67	8635.4
Agama gracilimembris	10	26.5	979	1124	26.7	1141.3	19.1	658.0	0.722	0.585	3.18	7254.2
Agama hispida	140	39.1	1204	827	18.2	372.7	26.0	175.2	0.665	0.212	4.83	6318.2
Agama impalearis	56	45.7	859	454	19.6	300.4	30.1	134.0	0.659	0.295	13.60	4174.3
Agama insularis	3	24.7	1212	2035	26.1	2380.0	17.5	1499.3	0.709	0.737	11.31	57270.1
Agama kaimosae	7	24.6	987	574	19.3	1089.4	16.1	313.9	0.656	0.547	9.22	8630.8
Agama lionotus	64	32.8	1222	681	23.0	664.0	17.3	274.6	0.529	0.403	8.38	4267.0
Agama mwanzae	22	24.1	593	450	19.9	887.0	15.2	312.3	0.632	0.694	3.02	789.8
Agama paragama	11	27.4	2449	1343	25.0	1159.5	20.3	670.5	0.743	0.499	8.86	28297.4

Agama planiceps	200	33.5	1306	654	21.8	442.7	19.3	235.3	0.576	0.360	12.35	6823.7
Agama rueppelli	33	27.8	602	283	26.4	338.0	16.5	131.1	0.594	0.463	7.11	1350.1
Agama sankaranica	44	28.6	1321	1141	26.4	1155.0	19.0	625.2	0.664	0.548	2.40	8127.1
Agama spinosa	10	31.3	440	187	23.6	249.5	21.6	64.9	0.691	0.347	12.80	3079.5
Agama weidholzi	28	29.8	1384	1301	27.1	1050.8	23.5	748.3	0.788	0.575	1.08	6252.6
Amphibolurus muricatus	2512	44.5	1724	719	15.0	1024.0	23.5	180.3	0.528	0.251	2.95	4068.5
Amphibolurus norrisi	259	30.3	544	289	15.8	415.8	23.6	95.6	0.778	0.331	0.33	544.1
Aphaniotis fusca	12	16	2185	1417	25.8	2749.2	9.5	595.2	0.596	0.420	2.82	34185.8
Bronchocela cristatella	168	21	3289	1893	25.9	2661.5	10.1	530.3	0.481	0.280	3.22	35356.2
Bufoniceps laungwalaensis	2	35.2	10	98	26.6	128.0	34.9	96.0	0.990	0.980	0.01	2.0
Calotes calotes	21	25.2	2701	1199	24.0	2556.4	11.6	604.5	0.461	0.504	11.17	30430.3
Calotes ceylonensis	6	14.3	758	952	26.1	1843.0	11.6	734.0	0.810	0.771	1.27	6590.8
Calotes chincollium	80	33.3	3495	3379	21.1	2056.1	19.7	1217.3	0.593	0.360	9.24	104182.1
Calotes emma	152	32	4349	3492	25.0	2664.5	17.2	1440.2	0.537	0.412	6.53	86670.2
Calotes htunwini	45	27.5	735	736	26.1	961.2	23.9	466.9	0.870	0.634	1.00	2998.4
Calotes irawadi	70	33.9	1616	1444	24.4	1393.1	23.5	740.0	0.692	0.512	5.27	38158.1
Calotes liocephalus	4	17.7	920	907	20.5	2368.5	13.0	657.0	0.733	0.724	3.87	15432.2
Calotes liolepis	20	18.2	2511	1240	25.1	2605.4	10.7	620.5	0.588	0.500	3.53	16366.1
Calotes mystaceus	288	37.9	4207	3281	24.5	1519.1	22.5	823.3	0.593	0.251	9.44	66525.1
Calotes nigrilabris	4	16.4	681	602	16.7	2269.5	14.5	449.5	0.881	0.747	0.90	2943.6
Calotes versicolor	653	45.6	5095	3593	24.6	1978.1	18.9	1061.8	0.415	0.296	10.56	132697.3
Ceratophora aspera	8	16.1	1789	1017	26.1	2951.0	9.7	598.3	0.603	0.588	1.75	14018.1
Ceratophora erdeleni	5	13.1	886	684	22.5	2821.6	10.7	499.8	0.815	0.731	0.91	7367.1
Ceratophora karu	5	13.3	984	706	22.5	2821.8	10.7	480.2	0.808	0.680	1.49	10802.2
Ceratophora stoddartii	9	23.6	1188	762	18.8	2011.8	13.4	495.9	0.570	0.651	17.59	2952.0
Chelosania brunnea	66	27.9	1120	1109	27.4	1169.1	20.4	782.5	0.730	0.706	1.13	9623.1
Chlamydosaurus kingii	650	34.9	3282	1735	26.4	1324.7	18.9	792.0	0.541	0.456	4.29	15635.4

Cophotis ceylanica	7	17.2	147	438	16.0	2044.1	14.8	411.1	0.859	0.939	0.86	110.2
Cophotis dumbara	7	16.5	479	929	20.8	2489.3	12.8	773.4	0.777	0.833	1.55	2410.1
Coryphophylax subcristatus	6	10.3	41	1374	26.4	3056.7	9.7	1284.3	0.940	0.935	0.10	215.4
Ctenophorus adelaidensis	143	32.7	943	531	18.8	602.4	23.4	303.2	0.717	0.571	1.32	2898.6
Ctenophorus caudicinctus	1529	40.5	1416	1013	24.1	401.0	29.3	206.7	0.723	0.204	5.62	8184.6
Ctenophorus clayi	156	38.5	237	240	22.0	240.4	30.6	80.4	0.796	0.335	4.18	429.4
Ctenophorus cristatus	737	34.7	636	434	17.8	267.4	27.4	52.9	0.791	0.122	0.75	331.4
Ctenophorus decresii	370	36.8	886	398	15.6	475.4	26.4	113.6	0.718	0.285	0.88	2567.8
Ctenophorus femoralis	116	36.1	158	181	24.5	255.1	27.0	110.2	0.749	0.609	1.68	87.0
Ctenophorus fionni	314	33.4	597	284	17.0	340.0	24.2	91.1	0.725	0.321	1.15	1157.8
Ctenophorus fordi	1339	39	615	270	17.6	285.1	28.2	37.1	0.724	0.138	1.17	318.4
Ctenophorus gibba	135	34	54	58	20.9	155.7	31.7	25.3	0.933	0.437	0.08	10.4
Ctenophorus isolepis	2089	39.7	766	636	23.4	297.1	30.0	136.6	0.755	0.215	4.48	1623.4
Ctenophorus maculatus	484	39.1	883	552	19.2	345.9	25.4	129.5	0.649	0.235	5.38	891.8
Ctenophorus maculosus	64	33.7	57	60	21.0	159.9	31.6	28.3	0.939	0.472	0.35	18.8
Ctenophorus mckenziei	28	26.9	117	97	17.5	280.7	25.1	54.9	0.932	0.566	0.05	90.4
Ctenophorus nuchalis	2813	41.2	1449	987	22.2	279.2	30.5	105.3	0.740	0.107	3.80	1757.6
Ctenophorus ornatus	263	34.8	987	622	17.5	481.4	27.0	181.7	0.776	0.292	1.56	6720.5
Ctenophorus pictus	1882	40.1	1253	466	19.0	251.1	28.9	46.4	0.721	0.100	3.61	594.4
Ctenophorus reticulatus	780	38.5	915	675	21.2	255.3	29.3	91.7	0.761	0.136	3.68	736.2
Ctenophorus rufescens	73	36.9	436	223	19.4	274.3	32.4	59.9	0.878	0.269	0.86	199.5
Ctenophorus salinarum	173	34.5	296	192	18.2	288.5	28.1	60.8	0.815	0.316	1.67	314.7
Ctenophorus scutulatus	351	37.2	624	448	20.2	262.0	29.5	85.3	0.792	0.190	2.29	348.2
Ctenophorus tjantjalka	50	34.7	94	79	20.6	167.2	31.6	28.4	0.909	0.359	0.20	45.5
Ctenophorus vadnappa	194	37.5	417	241	18.4	245.5	30.9	34.8	0.824	0.145	1.10	232.4
Diporiphora albilabris	226	29	1556	1141	27.0	1202.0	21.4	777.7	0.738	0.682	0.30	7204.7
Diporiphora amphiboluroides	70	36.9	229	220	22.3	265.6	30.6	99.9	0.829	0.454	3.09	372.6

Diporiphora arnhemica	95	28.9	870	828	27.1	787.7	24.7	523.1	0.854	0.632	0.48	5335.7
Diporiphora australis	407	37.2	2458	1529	22.4	993.5	22.2	481.4	0.596	0.315	4.30	17116.3
Diporiphora bennettii	346	30.4	1091	1037	27.3	1003.2	21.8	677.9	0.718	0.654	0.72	9745.6
Diporiphora bilineata	1966	40.2	2190	1406	26.9	1300.4	19.2	832.9	0.477	0.592	1.01	8978.3
Diporiphora lalliae	334	37.1	1363	1034	26.0	529.5	27.6	327.7	0.744	0.317	1.39	6359.1
Diporiphora linga	75	30	136	94	18.1	230.1	26.8	40.7	0.895	0.433	0.31	85.4
Diporiphora magna	937	35.1	1717	1187	27.1	923.9	23.2	612.4	0.661	0.516	0.95	7020.8
Diporiphora nobbi	1500	39.4	1739	1121	17.1	646.4	27.7	135.6	0.702	0.121	4.49	8945.0
Diporiphora pindan	139	32	589	550	26.8	485.3	23.9	345.5	0.746	0.628	0.42	3051.4
Diporiphora reginae	21	32	107	57	18.1	226.4	28.5	26.6	0.892	0.467	0.19	51.6
Diporiphora superba	35	25.6	956	1033	27.2	1142.6	19.3	806.1	0.752	0.780	0.36	9086.3
Diporiphora valens	38	35	191	226	24.5	352.9	29.9	179.2	0.854	0.793	0.58	237.9
Diporiphora winneckei	375	37.5	1680	1276	22.5	251.1	31.7	96.9	0.845	0.076	1.74	3014.9
Draco beccarii	120	12.9	972	769	26.5	2418.0	10.2	487.8	0.793	0.634	0.33	6599.2
Draco biaro	6	10.5	6	601	26.1	3065.8	9.8	577.3	0.929	0.961	0.10	11.7
Draco bimaculatus	16	18.3	2192	1193	25.1	2557.3	10.3	511.2	0.561	0.428	3.51	36629.9
Draco blanfordii	38	30.5	4586	3705	25.2	3067.3	17.4	1711.4	0.569	0.462	7.78	147960.1
Draco bourouniensis	2	8.9	143	1196	25.9	3045.5	8.1	1101.0	0.910	0.921	0.32	1458.0
Draco caerulhians	10	12.9	187	507	25.2	3577.9	9.9	445.1	0.768	0.878	0.71	329.6
Draco cornutus	8	14.1	2222	1319	26.3	3311.5	8.8	530.1	0.627	0.402	2.66	72549.4
Draco cyanopterus	5	17.5	569	625	24.8	2524.4	11.1	499.8	0.637	0.800	7.98	1996.8
Draco dussumieri	2	16.7	455	413	28.0	809.5	13.6	323.0	0.811	0.782	0.36	4050.0
Draco fimbriatus	21	17	1724	1459	25.4	3430.9	11.0	524.5	0.647	0.359	1.53	22585.1
Draco guentheri	6	12.2	336	530	26.1	1910.0	10.0	388.8	0.816	0.734	0.81	867.8
Draco haematopogon	32	17.8	2060	973	23.6	2791.3	10.8	496.0	0.605	0.510	4.80	25057.4
Draco indochinensis	6	21.4	1368	1618	22.8	2052.2	16.6	1048.2	0.773	0.648	5.72	22451.3
Draco lineatus	12	14.8	2111	1137	25.0	2248.5	9.6	541.0	0.650	0.476	2.18	25741.5

Draco maculatus	68	33.7	4671	3710	23.6	2094.9	19.2	1164.9	0.570	0.314	9.28	172003.0
Draco maximus	9	13.8	2356	901	25.2	3251.2	10.0	406.4	0.725	0.451	2.14	44234.1
Draco melanopogon	79	18.3	2026	1148	24.9	3000.9	11.0	456.7	0.603	0.398	2.62	20725.4
Draco mindanensis	6	14.3	1851	999	24.6	2558.7	10.2	509.7	0.712	0.510	1.86	35004.8
Draco obscurus	54	15.4	1615	1193	26.1	3366.4	10.3	513.2	0.672	0.430	1.13	9379.5
Draco ornatus	8	15.3	1309	1053	24.8	2749.4	10.1	518.5	0.659	0.492	2.74	42021.3
Draco palawanensis	11	12	713	766	26.6	1854.6	10.3	589.9	0.856	0.770	0.49	6039.3
Draco quadrasi	5	13.1	1386	1729	27.3	2550.4	10.4	1071.6	0.797	0.620	0.22	34908.9
Draco quinquefasciatus	25	15	2116	1513	26.3	3437.1	10.1	519.2	0.673	0.343	1.10	24264.9
Draco reticulatus	6	13.5	1004	830	25.7	2379.2	9.9	473.7	0.735	0.571	2.37	23371.5
Draco rhytisma	8	10.9	385	617	26.2	1916.1	9.8	492.8	0.900	0.799	0.12	1487.8
Draco spilonotus	46	16.5	1413	1357	24.7	2151.4	10.0	337.5	0.606	0.249	3.51	7258.6
Draco spilopterus	100	21.2	2803	2298	25.5	2368.9	10.3	722.5	0.486	0.314	4.31	39748.1
Draco taeniopterus	35	24	4586	3703	25.4	2920.9	17.0	1614.4	0.707	0.436	2.78	178610.5
Draco timorensis	4	13.3	855	872	23.4	1491.0	11.1	723.3	0.831	0.829	1.08	2691.2
Draco volans	43	19.7	3188	1907	25.5	2353.7	11.0	669.5	0.558	0.351	3.70	55480.8
Gonocephalus chamaeleontinus	19	21.8	1870	1183	24.9	3231.2	10.7	619.8	0.491	0.524	11.65	14889.9
Gonocephalus grandis	39	21.1	2906	1546	25.8	3044.1	10.5	511.3	0.498	0.331	1.92	20931.2
Gonocephalus kuhlii	4	18.4	1004	1003	20.1	2926.5	12.2	753.8	0.663	0.751	6.74	16036.2
Gowidon longirostris	1192	39.9	968	554	22.8	295.6	30.8	118.8	0.772	0.214	4.63	905.8
Gowidon temporalis	532	34.9	3743	1297	27.0	1536.5	16.2	893.5	0.466	0.689	0.59	8890.2
Hydrosaurus amboinensis	12	15	1063	882	25.8	2103.9	9.2	389.1	0.610	0.441	2.19	5353.3
Hypsilurus bruijnii	8	17.5	2725	948	23.7	2936.3	9.3	285.9	0.530	0.302	5.46	88235.7
Hypsilurus modestus	121	18.8	5960	2713	24.6	3498.0	10.2	468.7	0.542	0.173	3.83	123676.5
Hypsilurus nigrigularis	31	24.2	2718	1060	20.0	2416.4	12.3	455.1	0.510	0.429	12.59	19459.8
Hypsilurus papuensis	59	19.5	3167	1365	23.9	2687.1	11.3	491.0	0.580	0.360	6.93	42003.4
Intellagama lesueurii	3082	40.4	6141	1909	16.8	1241.5	22.4	286.0	0.555	0.150	3.48	10598.5

Japalura flaviceps	4	39	576	626	10.4	866.8	28.8	453.0	0.738	0.724	20.97	4995.6
Japalura polygonata	35	21	972	646	22.1	2250.2	17.9	374.6	0.853	0.580	0.78	2893.9
Japalura splendida	8	31.4	965	896	17.1	1340.0	24.6	678.4	0.784	0.757	2.81	11473.0
Japalura tricarinata	2	24.3	1037	1775	15.2	2068.5	23.0	1374.0	0.944	0.774	0.78	68820.5
Laudakia nupta	251	59.4	768	467	18.7	271.3	37.6	152.5	0.632	0.327	18.22	1252.1
Laudakia sacra	3	36.7	422	434	6.7	475.7	31.0	297.7	0.845	0.686	12.38	3583.0
Laudakia tuberculata	6	30.8	1086	1512	13.4	1842.2	23.6	1039.0	0.765	0.687	4.86	36869.4
Leiolepis belliana	64	29.1	4974	3713	26.1	1725.8	18.3	780.2	0.627	0.210	2.63	103847.1
Leiolepis guentherpetersi	3	16.9	483	1332	25.5	2299.3	15.8	1236.0	0.933	0.928	0.31	3689.3
Leiolepis guttata	2	17.6	851	1399	23.4	3108.5	12.7	920.5	0.722	0.658	12.01	4465.1
Leiolepis reevesii	5	20.4	884	825	24.4	1456.8	18.5	586.2	0.907	0.711	0.33	14083.9
Lophognathus gilberti	1871	37.8	1689	1128	26.4	857.7	23.9	552.5	0.632	0.490	2.84	14942.2
Lophosaurus boydii	59	26.5	3144	1928	21.8	1911.7	17.7	970.2	0.668	0.503	3.35	40196.4
Lophosaurus dilophus	64	17.1	5661	1984	25.4	3033.3	10.6	493.1	0.620	0.249	2.86	99890.2
Lophosaurus spinipes	317	36.5	1986	1201	16.5	1428.8	21.9	385.1	0.600	0.321	1.68	6523.2
Lyriocephalus scutatus	3	15	1482	853	25.1	3088.7	11.6	583.7	0.776	0.684	2.64	22857.3
Mantheyus phuwuanensis	8	24.1	1131	1835	23.2	2373.0	19.3	1406.8	0.801	0.767	4.15	22174.3
Moloch horridus	939	39.1	1517	1107	20.2	279.3	29.3	89.4	0.750	0.081	3.51	1551.1
Otocryptis wiegmanni	8	16	2114	1119	25.1	2780.1	11.4	619.9	0.713	0.554	1.83	24184.4
Paralaudakia caucasia	290	62.9	1020	502	11.5	323.0	38.3	125.6	0.608	0.250	11.44	1679.8
Paralaudakia erythrogaster	18	45.6	175	213	13.3	259.6	38.1	134.8	0.835	0.633	3.54	109.3
Paralaudakia himalayana	7	40.2	72	333	7.7	440.3	34.3	284.9	0.854	0.855	1.43	517.9
Paralaudakia lehmanni	4	43.8	469	383	12.8	514.3	37.5	272.8	0.855	0.712	9.87	4155.2
Paralaudakia microlepis	41	54.7	260	194	13.0	207.6	38.9	110.0	0.711	0.567	12.28	314.6
Paralaudakia stoliczkana	15	61.6	102	75	7.7	71.8	48.4	37.2	0.785	0.496	13.63	71.4
Phoxophrys nigrilabris	9	14.5	959	1345	25.5	3615.9	9.4	740.8	0.646	0.551	3.90	16822.7
Phrynocephalus axillaris	27	57.6	101	68	10.9	59.0	46.4	24.3	0.805	0.357	2.45	40.2

Phrynocephalus forsythii	10	50.2	54	47	10.5	61.4	46.0	29.3	0.916	0.623	2.34	38.3
Phrynocephalus guttatus	27	58.9	258	164	5.8	284.0	47.6	54.3	0.808	0.331	6.59	219.4
Phrynocephalus helioscopus	81	65.3	384	199	11.0	220.0	42.9	63.0	0.657	0.316	22.54	623.1
Phrynocephalus interscapularis	22	44.3	217	192	16.1	192.7	40.5	98.5	0.913	0.513	0.69	317.8
Phrynocephalus mystaceus	46	59.6	465	248	13.8	214.1	41.6	77.0	0.697	0.311	23.50	788.5
Phrynocephalus przewalskii	13	53.4	247	262	8.4	214.5	45.1	123.6	0.844	0.472	1.29	507.0
Phrynocephalus putjatai	6	46.1	144	254	2.6	391.5	38.7	224.8	0.840	0.885	6.33	204.4
Phrynocephalus raddei	9	42	119	153	16.6	198.2	40.0	107.8	0.952	0.704	0.35	147.1
Phrynocephalus scutellatus	197	58.3	396	216	17.0	148.0	38.8	78.2	0.665	0.362	10.64	271.8
Phrynocephalus theobaldi	22	45.1	538	434	4.3	382.5	33.8	276.8	0.750	0.638	15.56	991.7
Phrynocephalus versicolor	85	65.3	353	156	4.2	98.7	50.1	55.5	0.767	0.356	8.64	215.6
Phrynocephalus vlangalii	25	50	677	366	1.4	323.4	39.2	178.6	0.785	0.488	7.98	2922.1
Physignathus cocincinus	32	28	1811	1573	23.2	1940.7	17.5	998.8	0.625	0.635	6.51	15173.6
Pogona barbata	2571	41.4	1691	832	17.1	814.8	25.3	177.9	0.610	0.214	2.77	7585.1
Pogona henrylawsoni	44	31.5	342	524	24.5	483.9	28.7	264.7	0.910	0.505	0.96	1238.2
Pogona minima	21	32	860	509	18.2	523.4	25.0	226.0	0.783	0.444	2.53	7268.8
Pogona minor	1421	39.4	897	573	20.0	345.0	27.6	133.3	0.701	0.233	5.09	3333.8
Pogona nullarbor	72	29.1	131	82	17.7	240.1	24.9	35.5	0.857	0.432	0.50	92.2
Pogona vitticeps	2439	37.9	619	556	19.6	284.1	29.9	59.8	0.789	0.108	3.08	798.9
Pseudocalotes brevipes	5	24.6	98	1001	21.4	1740.6	20.6	950.6	0.837	0.950	3.16	216.6
Pseudocalotes flavigula	5	14.9	271	507	24.6	2984.0	11.9	453.2	0.800	0.894	1.47	546.4
Pseudocalotes kakhienensis	8	29.4	1000	1284	16.7	1573.8	23.7	822.0	0.807	0.640	2.78	7400.4
Pseudotrapelus sinaitus	134	42.8	349	222	22.0	91.7	27.4	51.1	0.640	0.230	7.27	470.1
Ptyctolaemus collicristatus	18	29	1186	1386	18.1	1852.6	19.4	1001.0	0.668	0.722	4.80	7235.8
Ptyctolaemus gularis	22	29.8	1774	2056	21.5	2604.9	22.9	1570.8	0.769	0.764	3.25	35650.2
Rankinia diemensis	997	39.1	1419	637	13.3	1173.1	23.2	208.7	0.594	0.328	3.93	3859.0
Saara asmussi	22	49.5	254	183	21.8	102.8	37.2	63.1	0.752	0.345	8.49	247.4

Saara hardwickii	5	35	2204	1970	26.4	985.2	25.8	769.8	0.738	0.391	3.08	136707.2
Saara loricata	42	46.1	251	271	24.3	304.2	36.5	186.9	0.791	0.690	2.26	380.4
Sitana ponticeriana	14	28.7	1248	1498	26.5	1134.3	17.5	678.0	0.610	0.453	2.85	19649.1
Stellagama stellio	481	51.8	1161	633	18.3	514.0	26.0	308.3	0.501	0.487	6.07	5647.7
Trapelus agilis	432	60.3	744	328	18.4	188.0	37.8	104.8	0.627	0.319	17.13	928.4
Trapelus flavimaculatus	14	37.2	240	163	25.7	99.6	26.8	60.8	0.719	0.373	8.82	424.1
Trapelus mutabilis	47	43.2	533	356	21.1	117.3	29.1	68.6	0.674	0.193	3.90	823.1
Trapelus ruderatus	148	59	552	326	16.4	313.5	38.5	160.1	0.652	0.491	27.31	665.5
Trapelus sanguinolentus	47	60	449	248	12.3	259.3	41.2	85.6	0.686	0.345	24.04	813.7
Trapelus savignii	33	29.6	501	346	19.6	177.1	24.5	110.0	0.827	0.318	0.40	1063.0
Tympanocryptis cephalus	225	38.2	740	653	23.1	308.3	29.9	139.4	0.782	0.213	3.89	1861.1
Tympanocryptis intima	458	36.5	645	553	21.3	184.6	31.5	47.4	0.862	0.086	0.94	755.1
Tympanocryptis lineata	840	42.3	1617	1071	21.7	363.1	28.5	153.9	0.674	0.144	8.00	4677.1
Tympanocryptis pinguicolla	72	40.8	548	322	14.6	548.1	26.8	83.7	0.656	0.260	17.38	1239.1
Tympanocryptis tetraporophora	1117	39.4	2617	1554	20.5	254.7	30.5	64.8	0.774	0.042	2.80	2179.6
Tympanocryptis uniformis	7	27.8	1048	1103	27.1	833.3	24.9	544.9	0.897	0.494	0.27	15583.2
Uromastyx acanthinura	6	39.1	186	144	18.9	214.0	32.4	76.3	0.829	0.530	0.53	309.1
Uromastyx aegyptia	234	40.9	436	288	22.1	64.6	28.5	38.2	0.698	0.133	2.79	207.6
Uromastyx benti	3	29.3	55	62	24.4	96.7	19.7	42.3	0.672	0.683	18.82	76.8
Uromastyx dispar	6	31	123	110	27.6	65.7	27.7	50.0	0.894	0.455	1.39	315.7
Uromastyx geyri	9	41.8	94	100	24.4	67.9	31.2	44.6	0.745	0.446	15.43	320.4
Uromastyx macfadyeni	3	22.8	54	34	28.2	44.0	21.0	19.0	0.921	0.559	0.99	50.3
Uromastyx ocellata	5	25.1	77	102	28.5	150.8	21.3	55.2	0.850	0.541	1.62	84.3
Uromastyx ornata	94	36.2	177	109	22.3	37.1	28.6	21.0	0.791	0.193	1.81	95.8
Uromastyx princeps	16	27.7	104	67	27.1	46.3	19.9	25.4	0.720	0.380	3.93	83.5
Uromastyx thomasi	9	28.2	15	28	26.4	40.4	19.8	22.4	0.703	0.802	0.23	2.8
Xenagama taylori	3	21.3	144	189	22.6	362.7	19.8	160.7	0.928	0.850	0.70	172.0

1: $PNB = Bio12_{max} – Bio12_{min}$
2: $PNB = Bio16_{max} – Bio17_{min}$
